# Co-Infection Patterns in Individual *Ixodes scapularis* Ticks Reveal Associations between Viral, Eukaryotic and Bacterial Microorganisms

**DOI:** 10.3390/v10070388

**Published:** 2018-07-22

**Authors:** Shaun T. Cross, Marylee L. Kapuscinski, Jacquelyn Perino, Bernadette L. Maertens, James Weger-Lucarelli, Gregory D. Ebel, Mark D. Stenglein

**Affiliations:** 1 Department of Microbiology, Immunology, and Pathology, College of Veterinary Medicine and Biomedical Sciences, Colorado State University, Fort Collins, CO 80523, USA; shaun.cross@colostate.edu (S.T.C.); mllayton@rams.colostate.edu (M.L.K.); jacquelynh88@gmail.com (J.P.); blmaertens@gmail.com (B.L.M.); james.weger@gmail.com (J.W.-L.); Gregory.Ebel@colostate.edu (G.D.E.); 2 Department of Biomedical Sciences and Pathobiology, Virginia Polytechnic Institute and State University, Blacksburg, VA 24061, USA

**Keywords:** *Ixodes scapularis*, Lyme disease, ticks, vector, metagenomics, tick-borne disease, co-infection, microbiome, virome, microbiota, South Bay virus, Blacklegged tick phlebovirus, bunyavirus, *Borrelia burgdorferi*, blacklegged tick, mutualism

## Abstract

*Ixodes scapularis* ticks harbor a variety of microorganisms, including eukaryotes, bacteria and viruses. Some of these can be transmitted to and cause disease in humans and other vertebrates. Others are not pathogenic, but may impact the ability of the tick to harbor and transmit pathogens. A growing number of studies have examined the influence of bacteria on tick vector competence but the influence of the tick virome remains less clear, despite a surge in the discovery of tick-associated viruses. In this study, we performed shotgun RNA sequencing on 112 individual adult *I. scapularis* collected in Wisconsin, USA. We characterized the abundance, prevalence and co-infection rates of viruses, bacteria and eukaryotic microorganisms. We identified pairs of tick-infecting microorganisms whose observed co-infection rates were higher or lower than would be expected, or whose RNA levels were positively correlated in co-infected ticks. Many of these co-occurrence and correlation relationships involved two bunyaviruses, South Bay virus and blacklegged tick phlebovirus-1. These viruses were also the most prevalent microorganisms in the ticks we sampled, and had the highest average RNA levels. Evidence of associations between microbes included a positive correlation between RNA levels of South Bay virus and *Borrelia burgdorferi*, the Lyme disease agent. These findings contribute to the rationale for experimental studies on the impact of viruses on tick biology and vector competence.

## 1. Introduction

*Ixodes scapularis*, the blacklegged or deer tick, is the main North American vector for *Borrelia burgdorferi*, the causative agent of Lyme disease. In the USA, there are an estimated 300,000 cases of Lyme disease per year, and the incidence of tick-borne diseases is increasing [1,2,3]. In addition to *B. burgdorferi*, *I. scapularis *ticks harbor other pathogens, including eukaryotic (*Babesia microti*), bacterial (*Anaplasma phagocytophilum*, *B. mayonii*, *B. miyamotoi* and *Ehrlichia muris eauclarensis*), and viral (Powassan virus) agents [4,5,6,7,8]. It is possible that individual ticks can be co-infected by more than one of these pathogens, and co-infection of a vertebrate can impact clinical outcome [9,10,11].

Ticks also harbor non-pathogenic microbes, and it has been recognized for some time that these have the potential to influence tick physiology and vector competence, the ability of the tick to acquire, harbor and transmit a pathogen [12,13,14,15,16,17,18,19,20,21,22]. For example, *Amblyomma americanum *ticks dysbiosed by antibiotic injection exhibited a marked decrease in reproductive success [21]. In *I. scapularis* larvae with decreased bacterial loads, *B. burgdorferi* colonization of the midgut was less efficient [22]. Also, *Anaplasma marginale* levels were lower in *Amblyomma americanum *ticks with altered microbiomes [20]. As in all organisms, it is clear that tick-associated microbiota can exert a significant effect on their host. 

Metagenomic studies have also recently identified a number of new tick-associated viruses in the northeastern USA, in several European countries, and in China [23,24,25,26,27,28,29,30]. Two groups of bunyaviruses seem to be particularly common in *Ixodes* ticks: a lineage that includes South Bay virus in *I. scapularis *in the USA and Grotenhout virus in *I. ricinus *in Europe, and a lineage that includes the blacklegged tick phleboviruses in American *I. scapularis* and Norway phlebovirus in European *I. ricinus* [24,28,30]. Yet the biological impact of these viruses remains largely unknown. And previous studies have for the most part characterized the bacterial and viral microbiomes of ticks separately [14,15,16,24,27,30,31,32,33].

Therefore, to understand the possible influence of nonpathogenic viral components of the microbiota of *I. scapularis*, we simultaneously measured RNA levels of eukaryotic, bacterial and viral microbes in or on 112 individual adult ticks collected from Wisconsin, USA. This is an area of high Lyme disease risk, and the microbiome of *I. scapularis *from this region has not been evaluated in this manner [34]. We identified known microorganisms, including pathogens, as well as new virus-like sequences and a previously undescribed filarial worm. We characterized the prevalence, abundance and co-infection rates of microorganisms, and identified statistically significant co-occurrence and correlation patterns between microbiome constituents. We found that, as in other *I. scapularis* populations, South Bay virus and blacklegged tick phleboviruses were particularly common in these ticks [24,27,30]. These viruses were also involved in the majority of statistically significant associations with other microbes, including with *B. burgdorferi*.

## 2. Materials and Methods

### 2.1. Sample Collection

Adult *I. scapularis* were collected near Spooner Wisconsin by dragging in October 2015. Adult ticks were transported to the laboratory, identified to species and stored in individual cryovials in mosquito diluent (20% FBS, 1× PBS, 1× Penicillin/Streptomycin) at −80 °C until further processing. Ticks were not surface cleaned, so we sampled microorganisms present on the surface of ticks as well as those contained within ticks. Remaining tick halves were stored in this preservation medium for future possible virus isolation; 61 female and 51 male ticks were analyzed.

### 2.2. RNA Extraction

Ticks were sliced down the sagittal plane using a sterilized blade. One half of the tick was added to a 2 mL centrifuge tube along with a single sterile ball bearing, and 1 mL TRIzol (Ambion Life Technologies, Thermo Fisher Scientific, Waltham, MA, USA); the other half was added to fresh mosquito diluent and stored at −80 °C to be used for future analysis. The tick half in TRIzol was homogenized in a TissueLyzer II (Qiagen, Hilden, Germany) at 30 Hz for 4 min. 200 μL of chloroform (SigmaAldrich, St. Louis, MO, USA) was added, shaken by hand for 15 s, and incubated at room temperature (RT) for 2 min. RNA was further purified using RNA Clean and Concentrator-5 spin columns (Zymo, Irvine, CA, USA) as described [35]. RNA was quantified fluorometrically and stored at −80 °C.

### 2.3. Shotgun Metagenomic Library Preparation

Shotgun metagenomic libraries were prepared from total tick RNA as follows. 5 μL of RNA was added to 200 pmol of a random pentadecamer oligonucleotide and incubated for 5 min at 65 °C. Following incubation, the mixture was set on ice for 1 min. A reverse transcription mixture containing the following was added (12 μL reaction volume): 1× SuperScript III (SSIII) FS reaction buffer (Invitrogen, Carlsbad, CA, USA), 5 mM dithiothreitol (Invitrogen), 1 mM each deoxynucleotide triphosphates (dNTPs) (NEB), and 100 U SSIII reverse transcriptase enzyme (Invitrogen). The RNA-oligomer with the reverse transcription mixture was incubated at 42 °C for 30 min, then at 50 °C for 30 min, then at 70 °C for 15 min. Total HeLa cell RNA and water were processed in parallel as controls. RNA templates were removed by adding a mixture 1 U RNase H (NEB) diluted in 160 pmol random pentadecamer and 5 μL 1× SSIII FS reaction buffer. Samples were incubated at 65 °C for 20 min followed by 94 °C for 2 min. This single-stranded cDNA was converted to double-stranded DNA by adding 2 mM each dNTPs, 1× SSIII FS reaction buffer, and 2.5 U Klenow DNA polymerase (3′ to 5′ exo-, NEB) and incubated at 37 °C for 15 min. The DNA was purified using SPRI (Solid Phase Reversible Immobilization) beads at a 1:1.5 DNA/beads ratio and eluted in 20 μL nuclease-free water (NFW). The dsDNA concentration was measured fluorometrically using a Qubit 3.0 fluorometer (Thermo Fisher Scientific, Waltham, MA, USA). DNA was tagmented by adding 10 ng of the dsDNA, 1× Tagment DNA buffer (Illumina, San Diego, CA, USA) and 0.5 μL 10× Nextera Tagment DNA enzyme (Illumina) at a final volume of 12 μL, followed by incubation at 55 °C for 10 min. Tagmented DNA was cleaned with SPRI beads and eluted in 15 μL NFW. The cleaned, tagmented DNA was used as a template (5.8 μL) for addition of full-length adapters with unique bar-code combinations via PCR. This PCR reaction (25 μL final volume) contained the following: 1× Kapa real-time library amplification mix (Kapa Biosystems, Roche, Basel, Switzerland), 0.33 μM each of the primers 5′-CAAGCAGAAGACGGCATACG-3′ (P1) and 5′-AATGATACGGCGACCACCGA-3′ (P2), and 0.02 μM each of adapter 1 and 2 bar-coded primers [36]. The PCR reaction was run at 72 °C for 3 min, 98 °C for 30 s, and 12 cycles of 98 °C for 10 s, 63 °C for 30 s, and 72 °C for 3 min. PCR reactions were cleaned using SPRI beads, eluted in 15 μL NFW, and concentrations were measured fluorometrically. Equal masses of DNA from each sample were pooled, cleaned using SPRI beads, and eluted in 60 μL of nuclease-free Tris EDTA pH 8.0 (TE). The pooled libraries were size selected (range of 350–500 nucleotides) using a BluePippin and a 2% agarose Pippin gel cassette (Sage Science, Beverly, MA, USA) according to manufacturer’s protocol. Size-selected pools were cleaned using SPRI beads with a 1:1.4 DNA/beads ratio and eluted in 20 μL NFW. Cleaned, selected pools were subjected to a final PCR containing 1x Kapa real-time amplification master mix (Kapa Biosystems), 500 pmol of both P1 and P2, and 10 μL of selected pools at a total volume of 50 μL. Thermocycling conditions were 98 °C for 45 s, followed by varying amounts of cycles of 98 °C for 10 s, 63 °C for 30 s, and 72 °C for 2 min. Number of cycles was determined by the amount needed for the fluorescence to pass Kapa standard 1. Amplified pools were cleaned using SPRI beads at a 1:1.5 DNA/beads ratio and eluted in 18 μL TE. Final library quantification was performed using the Illumina library quantification kit (Kapa Biosystems) according to manufacturer’s protocol. Libraries were sequenced using an Illumina NextSeq 500 instrument using paired-end 2 × 150 sequencing from a NextSeq 500/550 Mid Output Kit v2 (300 cycles) (Illumina).

### 2.4. Sequence Analysis

Metagenomic sequencing datasets were processed to taxonomically assign non-tick reads. First, low-quality sequences and adapter sequences were removed using the cutadapt tool version 1.14 under the following settings: -a AGATCGGAAGAGC -A AGATCGGAAGAGC -g GCTCTTCCGATCT -G GCTCTTCCGATCT -a AGATGTGTATAAGAGACAG -A AGATGTGTATAAGAGACAG -g CTGTCTCTTATACACATCT -G CTGTCTCTTATACACATCT, -q 30,30, --minimum-length 80, and -u 1 [37]. PCR duplicates were collapsed using the CD-HIT-EST tool version 4.7 with the -c 0.96 parameter [38]. Host tick sequences were removed using Bowtie2 version 2.3.2 [39]. First, a bowtie index was created using the reference genome of *I. scapularis *[40]. Reads were then removed using a local alignment with the parameters --local --sensitive --score-min C,60,0. SPAdes genome assembler version 3.10.1 [41] was used to generate contiguous sequences from the remaining reads. Contigs longer than 150 nucleotides (nt) were taxonomically categorized using the BLASTn alignment tool version 2.6.0+ [42,43]. Contigs were assigned taxonomically to the sequence with the highest alignment score and an expect value less than 10^−8^ [42,44]. In order to taxonomically assess reads that were too divergent to produce a high-scoring nt-nt alignment, DIAMOND version 0.9.9.110 was used to query the NCBI nr database with an expect value of 10^−3^ [45]. The number of reads aligning to individual taxa were tallied by remapping host-filtered reads to SPAdes contigs using bowtie. If a contig aligned equally to multiple taxa, the result was collapsed at the lowest common ancestor of the matches. For several genera of bacteria (*Wolbachia*, *Rickettsia* and *Ehrlichia*), it was difficult to assign contigs at the species level because they aligned equally well to sequences from two or more species with the genus. Rather than equally distributing these reads to the multiple species, and potentially assuming the presence of a species that may not actually be present, we collapsed reads that aligned to these taxa at the genus level. Phage sequences were detected at very low levels (≤17 reads in 6 of the datasets), and phage sequences were not further analyzed.

Virus-mapping contigs were collapsed when possible by de novo assembling contigs that aligned on a protein level to a particular virus in Geneious version 11.0.4 [46]. Gaps were filled using PCR and Sanger sequencing. Draft virus assemblies were validated by remapping reads using Bowtie2 as above. All sequencing datasets have been deposited in NCBI Sequence Read Archive (SRA) under BioProject accession PRJNA477560 [47].

### 2.5. Validation of Sequencing by PCR

PCR was used to validate sequencing results from a subset of random ticks that contained at least 4 co-infecting microorganisms. dsDNA remaining from library preparation (see above) was diluted 1:20 in nuclease-free water. Primers were created for viral sequences, while existing primers were used for *Borrelia burgdorferi*, *Anaplasma phagocytophilum*, *Babesia microti*, *Borrelia miyamotoi* [48], and the positive control *I. scapularis* glycerol-3-phosphate dehydrogenase (GPDH) primers [49] (Supplemental Table S1). PCR reactions contained: 1x Luna Universal qPCR Master Mix (NEB, Ipswich, MA, USA), 10 μM each of forward primer and reverse primer, and 5 μL of DNA template at a final volume of 20 μL. Thermocycling conditions for all microorganisms were 95 °C for 3 min, followed by 40 cycles of 95 °C for 10 s and 60 °C for 45 s. For gpdh, thermocycling conditions were 95 °C for 3 min, followed by 40 cycles of 95 °C for 10 s and 55 °C for 45 s.

### 2.6. Statistical Analysis of Microbial Relationships

To measure associations between microorganisms, a table describing the number of reads mapping to various taxa in individual ticks was imported into R studio version 1.0.153 [50]. Co-occurrence relationships were measured using the ‘cooccur’ package version 1.3 and the function cooccur [51]. This package uses presence–absence datapoints and a hypergeometric distribution to calculate the probability that one site (an individual tick) contains both species 1 and 2, and whether they occur more or less frequently than expected. Correlation measurements were performed using the ‘psych’ package version 1.7.8 and the function corr.test with a Pearson method and Bonferroni adjustment [52]. Correlations were only analyzed for ticks that were co-infected with both microbes being analyzed, and Pearson coefficients were only considered significant if the adjusted *p*-value was less than 0.05. Microorganism prevalence by tick sex was statistically assessed using a pair-wise Fisher’s exact test with a Bonferroni adjustment. R Code and data matrices are available in Github repository: https://github.com/scross92/coinfection_patterns_ixodes_scapularis. In mean abundance (RPM) calculations, values of 0 were set to NA in order to not factor into the reads per million (RPM) calculation.

### 2.7. Phylogenetic Analysis of Novel Microorganisms

For phylogenetic analysis of predicted viral sequences, the NCBI nr protein database was queried using the BLASTX tool, and aligning sequences with an expect value of less than 10^−3^ were downloaded [43]. Sequences were collapsed to a representative subset using the CD-HIT tool version 4.7 using parameter -c 0.9 [38]. These representative sequences were aligned using MAFFT version 7.310 under the --auto mode [53]. Alignments were trimmed with trimAl version 1.4.rev15 in the --strictplus mode [54]. These trimmed alignments were imported into Geneious version 11.0.4 and manually inspected. In case of partial sequences, alignments were trimmed to the length of partial sequence and any remaining sequences that were poorly aligned were removed. Phylogenetic trees were created from these alignments using PhyML version 3.3.20180109 under the LG mode and 100 bootstraps [55]. Phylogenetic trees were visualized using FigTree version 1.4.3 (http://tree.bio.ed.ac.uk/software/figtree/).

Phylogenetic analysis of the novel filarial worm sequence was performed essentially as previously described [56]. Primers were used to amplify the 12S rDNA sequence. The PCR product was Sanger sequenced, and the product was aligned against 12S rDNA sequences derived from other filarial worms using MAFFT version 7.310 under the L-INS-i mode. This alignment was then used to create a phylogenetic tree using PhyML using the HKY85 mode and 100 bootstraps.

## 3. Results

### 3.1. Taxonomic Assessment of the Ixodes Scapularis Microbiome

**Figure 1:**
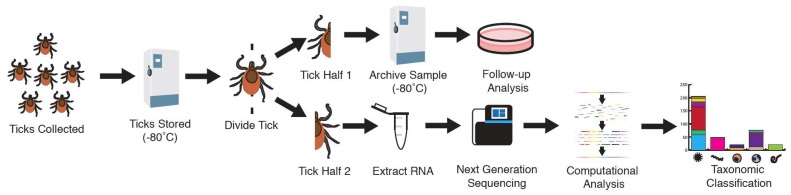
Tick analysis workflow. Adult *Ixodes scapularis* ticks were collected from northwest Wisconsin. Ticks were then stored at −80 °C in mosquito diluent. Individual ticks were divided in half. The first half was subjected to next-generation sequencing, computational analysis, and taxonomic classification. The second half was archived at −80 °C for future analysis.

In October 2015, 112 adult *Ixodes scapularis* (61 female; 51 male) were collected in northwest Wisconsin. Ticks were cut in half. One half was stored in mosquito diluent at −80 °C, and RNA was extracted from the other half (Figure 1). RNA-derived shotgun libraries were sequenced using paired-end 2 × 150 sequencing on an Illumina NextSeq instrument, generating an average of 2.1 × 10^6^ read pairs per dataset. After removing low-quality, adapter and tick-derived reads, an average of 4.8 × 10^4^ read pairs per dataset remained (2.7%). Remaining reads were assembled and taxonomically assigned by comparison at nucleotide and protein levels to NCBI database sequences. In most cases, contigs shared a high degree of sequence identity with existing sequences (Table 1). In other cases, contigs were less closely related to database sequences. For instance, we identified contigs that shared between 76.1% and 98.2% nt identity in BLASTN alignments with various nematode sequences (Table 1). Because these sequences appeared to derive from a previously uncharacterized worm, confident species or genus-level assignment was not possible, and contigs were assigned at the level of the family *Onchocercidae* (nematodes). Similarly, unambiguous species-level assignment was not possible for contigs mapping to certain bacterial taxa (*Rickettsia*, *Ehrlichia* and *Wolbachia) *so contigs mapping to these taxa were assigned at the genus level. We calculated the number of reads mapping to particular taxa per million unique reads (RPM) as a measure of RNA level and taxon abundance. Because we did not clean ticks prior to RNA extraction, it is possible that some sequences derived from microbes present on the surface of ticks. Also, detection of pathogen sequences does not necessarily indicate that that particular tick would be a competent vector for the pathogen. Total HeLa cell RNA and water were processed and analyzed in parallel as positive and negative controls.

**Figure 2:**
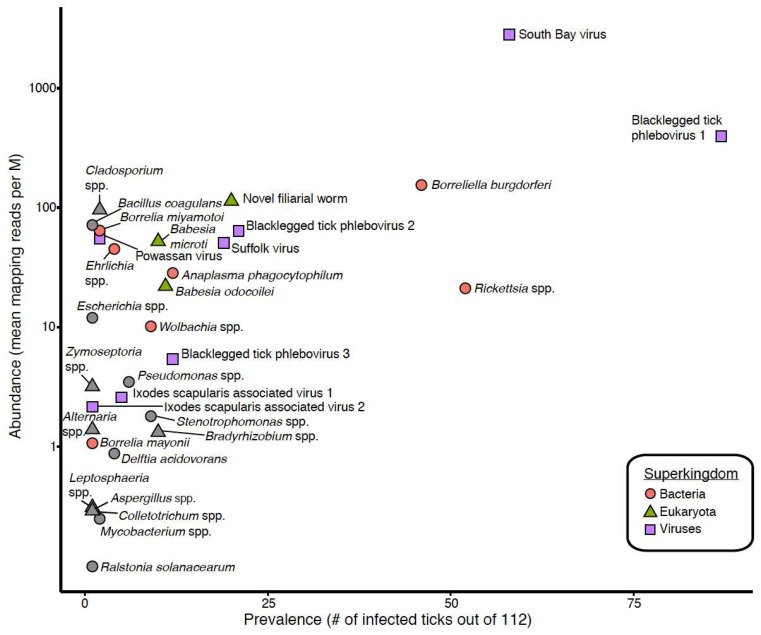
South Bay virus and Blacklegged tick phlebovirus are the most abundant and prevalent microorganisms in the sampled *I. scapularis*. The prevalence (number of infected ticks out of 112) and average RNA level (average mapping reads per million unique reads on a log scale) for the indicated taxa are plotted. The superkingdom of each taxa is indicated by shape and color as indicated. The 18 taxa that were selected for focused analysis are colored (other taxa in grey).

**Figure 3:**
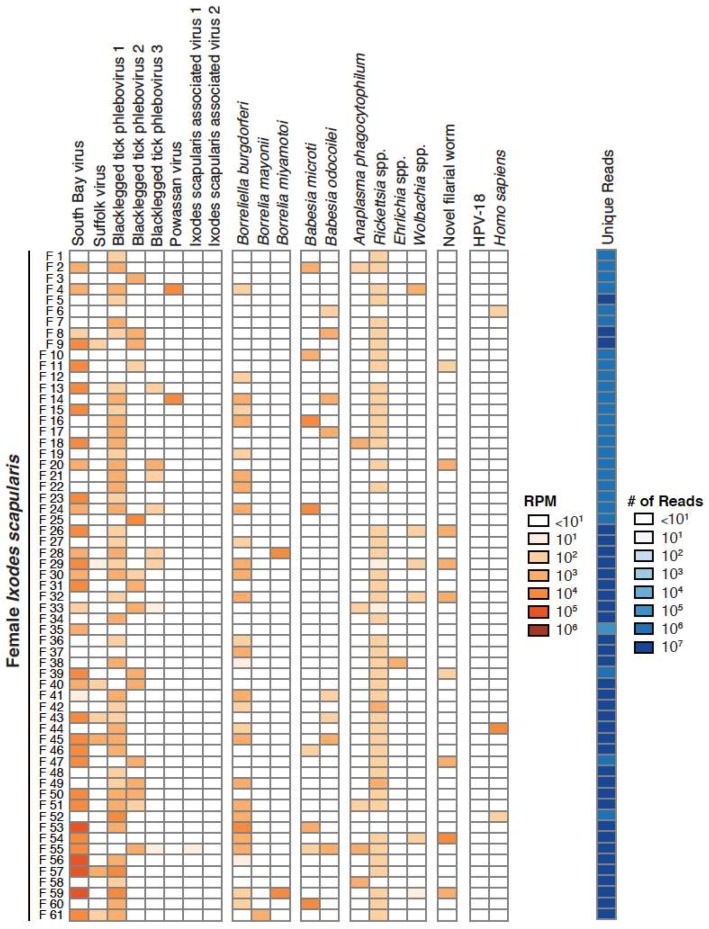
Abundance of predominant microbial constituents of female adult *Ixodes scapularis*. 18 taxa of interest were selected for abundance analysis. Between 1 and 9 taxa were detected in female adult ticks (*n* = 61). The number of mapping reads per million unique reads (RPM) is shown, as is the number of unique reads in each dataset. RPM values >10 are shown. HPV-18: human papillomavirus type 18.

**Figure 4:**
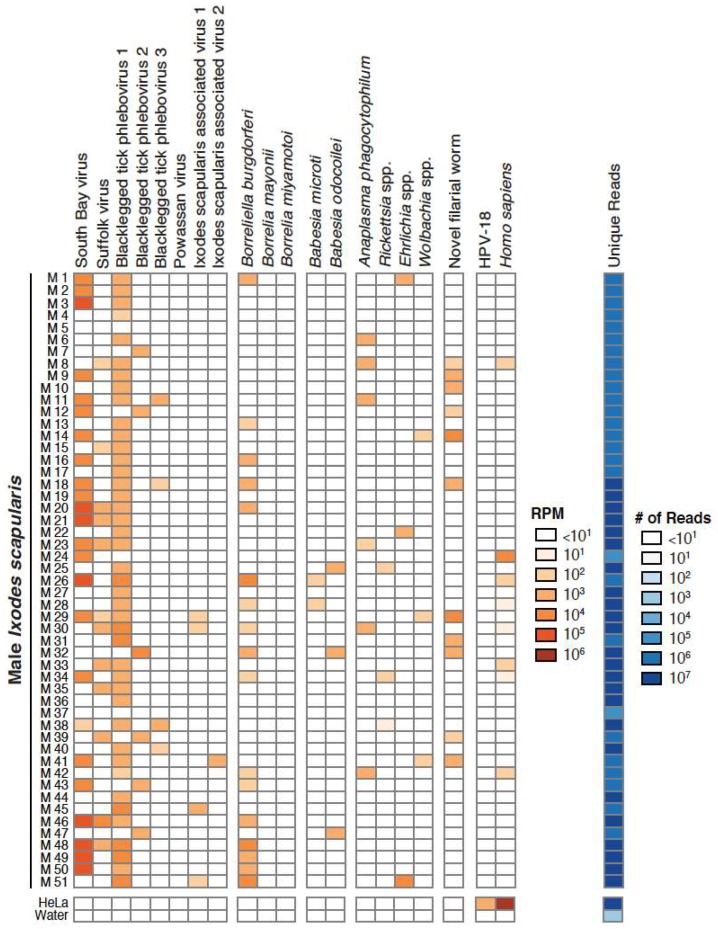
Abundance of predominant microbial constituents of male adult *Ixodes scapularis* and controls. 18 taxa of interest were selected for abundance analysis. In male adult ticks (*n* = 51), between 0 and 5 taxa were detected. The number of mapping reads per million unique reads (RPM) is shown, as is the number of unique reads in each dataset. RPM values >10 are shown. Control datasets generated from HeLa cell total RNA and water are shown. HPV-18: human papillomavirus type 18.

We focused our analyses on 18 taxa that accounted for 89% of the assigned non-tick reads in our datasets. These 18 taxa included South Bay virus, Suffolk virus, Blacklegged tick phleboviruses 1–3, Powassan virus, Ixodes scapularis associated viruses 1 and 2, *B. burgdorferi *sensu stricto, *B. mayonii*, *B. miyamotoi*, *Babesia* (*Ba.*) *microti*, *Ba. odocoilei*, *Anaplasma (A.) phagocytophilum*, *Rickettsia*, *Ehrlichia*, *Wolbachia* and *Onchocercidae*. These taxa were selected because they were the most abundant and prevalent in individual ticks and/or because they are human pathogens (Figure 2, Figure 3 and Figure 4; Table 1). Female ticks contained between 1 and 9 of these taxa (female 55) (Figure 3), while individual male ticks contained between 0 and 6 (male 5, male 29) (Figure 4). 

The most abundant and prevalent taxa in individual ticks were blacklegged tick phlebovirus 1 (BLTPV1) and South Bay virus (SoBV), with prevalences of 78% and 52% and mean mapping read levels of 395 and 2796 RPM (Figure 2, Table 1). *Rickettsia* species and *B. burgdorferi*, detected in 46% and 41% of ticks and with mean levels of 21 and 154 RPM, were the next most prevalent. 

We evaluated prevalence of taxa by sex and found that *Rickettsia* spp. were detected more often in female ticks (80.3%) than in males (5.9%; Table 1). This difference was the only statistically significant difference in taxon prevalence by sex (*p*-value = 3 × 10^−15^). This difference has been observed in previous studies [17,57,58,59]. It has been proposed that the higher prevalence in females may be attributed to an adaption of *Rickettsia *spp. to transovarial transmission [17].

### 3.2. Validation of Metagenomic Sequencing Results

**Figure 5:**
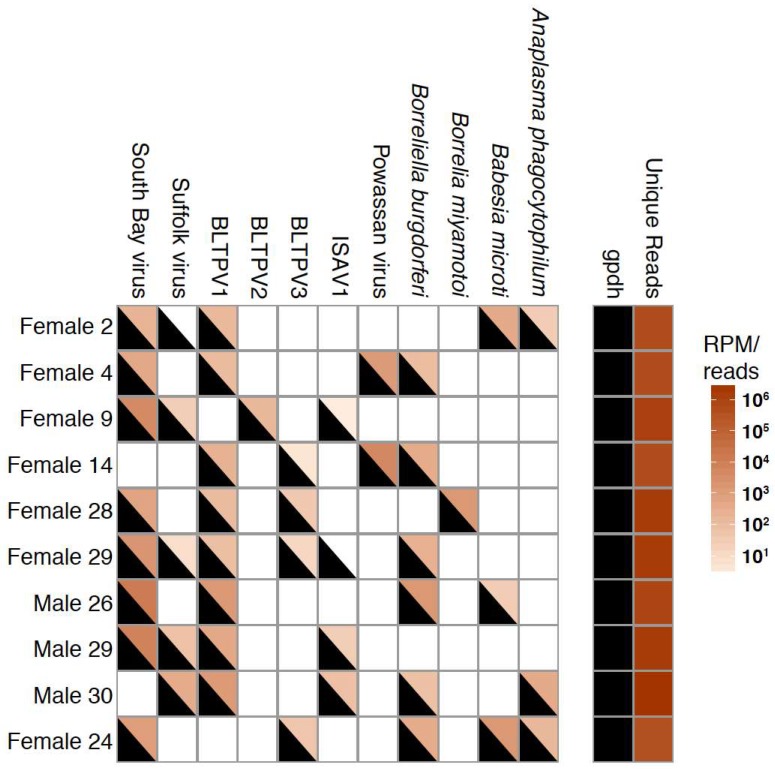
PCR detection of microbes was concordant with sequencing-based detection. Ten ticks with at least four organisms detected by sequencing were randomly selected for validation by RT-PCR. PCR positive samples are indicated by a black triangle. *I. scapularis* glycerol-3-phosphate dehydrogenase (gpdh) was used as a positive control for detection of tick RNA. The number of unique reads from each NGS dataset is shown using the same color scale as RPM values. BLTPV: Blacklegged tick phlebovirus; ISAV: Ixodes scapularis associated virus.

We used RT-PCR to corroborate sequencing results. Ten ticks that harbored at least 4 organisms were randomly selected. We performed RT-PCR using custom primers and previously published primers for *B. burgdorferi*, *A. phagocytophilum*, *Ba. microti*, and *B. miyamotoi* [48] (Supplemental Table S1). Primer to amplify *I. scapularis *glycerol-3-phosphate dehydrogenase (GPDH) mRNA were used as a positive control [49]. In all cases where an organism was detected by sequencing, it was also detected by PCR (Figure 5). However, there were two cases where an organism was detected by PCR but not sequencing: Suffolk virus in tick F2, and Ixodes scapularis associated virus 2 in tick F29 (Figure 5). We attributed this discrepancy to the fact that PCR is generally more sensitive than sequencing [60].

### 3.3. Detection of New Microorganisms

**Figure 6:**
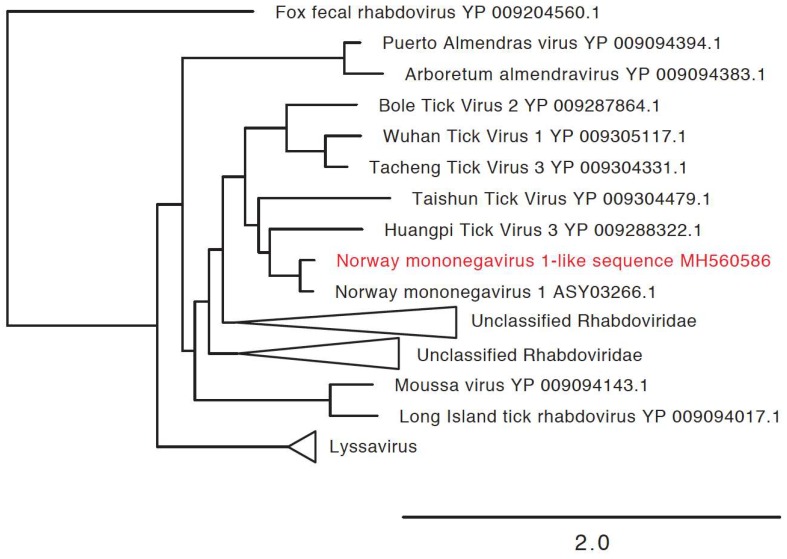
Phylogenetic characterization of new mononegavirus sequence. Phylogeny based on an alignment of a 508 amino acid region of the viral RNA dependent RNA polymerase (RdRp). This alignment includes mononegavirus reference sequences available through NCBI. Additional closely related unclassified viruses were also included. Triangles indicate collapsed clades. The novel virus sequence is shown in red. Heartland virus was used as an outgroup to root the tree.

**Figure 7:**
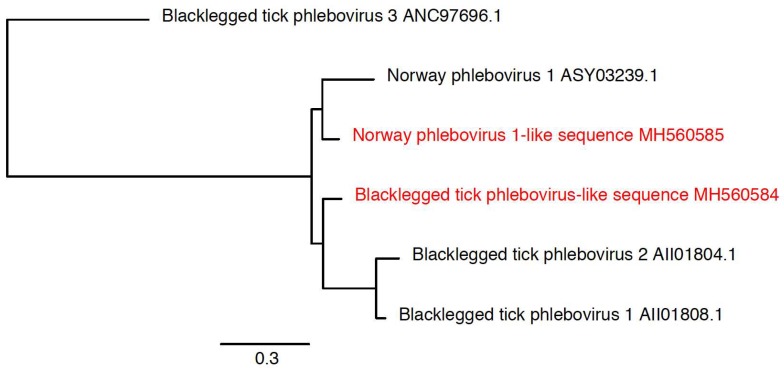
Phylogenetic characterization of novel phleboviruses. Phylogeny based on an alignment of a 176 amino acid region of the nucleocapsid protein. This alignment includes phlebovirus reference sequences available through NCBI. Additional closely related unclassified viruses were also included. The novel viruses are shown in red. Phasi Charoen-like phasivirus was used as an outgroup to root the tree.

We also identified new virus or virus-like sequences. These were at relatively low levels in relatively few ticks, and included a mononegavirus sequence most closely related to Norway mononegavirus 1 (Figure 6, Table 2), and sequences related to the S segments of Blacklegged tick phleboviruses and Norway phlebovirus 1 (Figure 7, Table 2). We did not identify phlebovirus L or M segment sequences, and cannot rule out the possibility that the S segment-like sequences correspond to endogenous viral elements. However, the S segment sequences had low coverage, and it may be that the L and M sequences were below the limit of detection. Virus scaffolds ranged from 791 nucleotides to 5020 nucleotides long, and no scaffold was coding complete.

**Figure 8:**
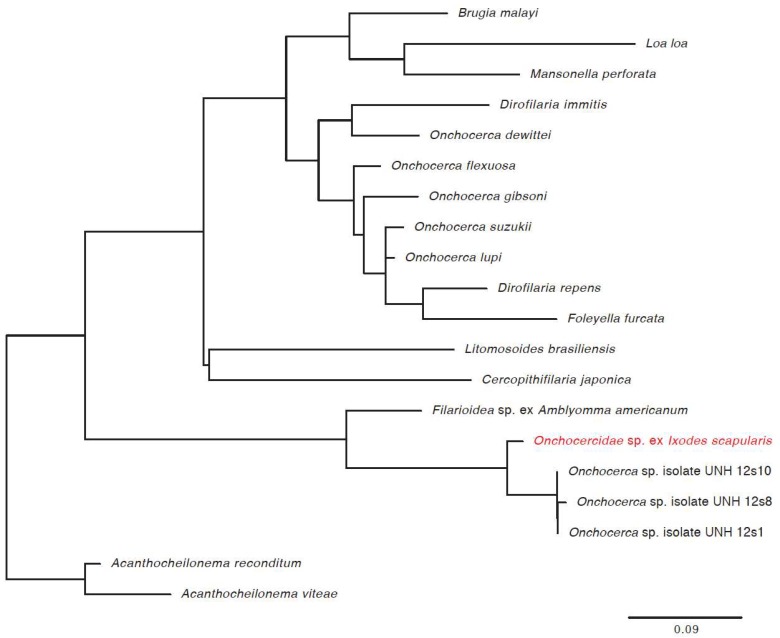
Phylogenetic characterization of novel filarial worm. Phylogeny based on an alignment of a 174 base pair region of the 12S rDNA sequence. This alignment included other filarial worm 12S rDNA sequences as used in Namrata, et al. [56]. The novel filarial worm (*Onchocercidae* sp. ex. *Ixodes scapularis*) is shown in red.

We also characterized the phylogenetic placement of the filarial worm (*Onchocercidae* sp. ex *Ixodes scapularis*) that we identified in 20 of the ticks. We used PCR and Sanger sequencing to determine the worm 12S rRNA gene sequence from 6 positive samples and found them to share ≥98.5% pairwise nt identity. A tree based on the alignment of these sequences with related nematode sequences showed them to cluster most closely to other filarial worms recently found in other *I. scapularis* (Figure 8) [56].

### 3.4. Co-Occurrence and Correlation Analyses

We next searched for evidence of associations between members of the microbiome of these *I. scapularis *by examining patterns of co-infection. We first evaluated whether pairs of taxa co-occurred more or less than would be expected given their individual infection rates. If co-infection was found more often than expected (positive co-occurrence), it could suggest that infection by one organism could predispose to infection by the second, or that ticks are more likely to acquire both organisms from feeding on a co-infected vertebrate. If co-infection was found less often than expected (negative co-occurrence), it could suggest that infection by one organism prevents infection by the second.

**Figure 9:**
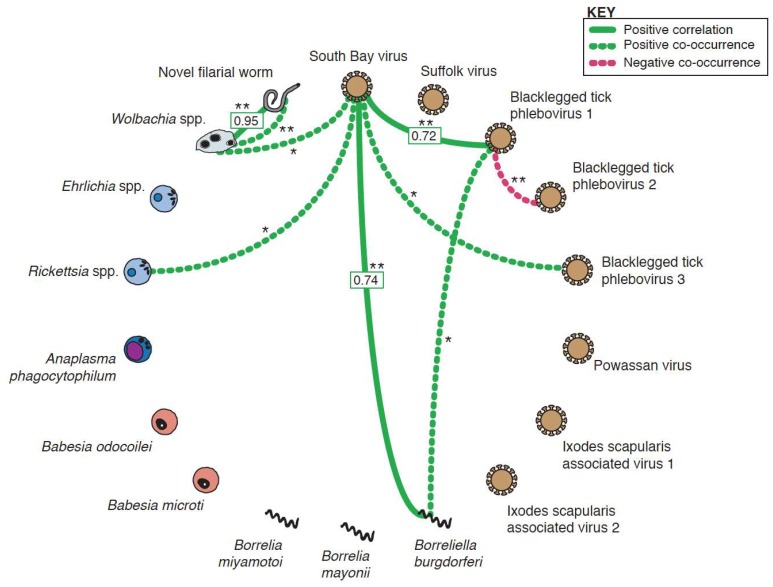
Co-occurrence and correlation relationships among tick microbiota. Statistically significant co-occurrence and correlation relationships between microbial constituents within *Ixodes scapularis* ticks. Positive co-occurrence or correlations are depicted in green, while negative relationships are depicted in magenta. Correlation coefficients are shown in boxes. Corrected *p*-values are indicated by: * < 0.05; ** < 0.01.

Five positive and one negative co-occurrence relationships were identified after correcting for multiple hypothesis testing (Figure 9). The positive relationships were: *Wolbachia* spp. and the novel filarial worm (*p*-value < 1 × 10^−6^), *Wolbachia* spp. and SoBV (*p*-value = 0.021), *Rickettsia* spp. and SoBV (*p*-value = 0.041), SoBV and BLTPV3 (*p*-value = 0.02), and Blacklegged tick phlebovirus 1 (BLTPV1) and *B. burgdorferi *(*p*-value = 0.039). The sole negative co-occurrence was between BLTPV1 and BLTPV2 (*p*-value < 1.0 × 10^−6^) (Figure 3 and Figure 4). 

We then tested whether the abundance of taxa were correlated within individual co-infected ticks, which could suggest that infection by one organism impacts replication of another. After a Bonferroni multiple testing adjustment, three statistically significant positive correlations were identified (Figure 9, Supplemental Figure S1). These were *Wolbachia* spp. and the filarial worm (Pearson coefficient = 0.96; *p*-value = 8.4 × 10^−3^), SoBV and *B. burgdorferi* (Pearson coefficient = 0.75; *p*-value = 1.4 × 10^−3^), and SoBV and BLTPV1 (Pearson coefficient = 0.72; *p*-value = 1.25 × 10^−6^).

## 4. Discussion

Recent studies have made significant inroads characterizing the microbiome of ticks, which is a rich mixture of viruses, eukaryotes and bacteria [14,15,16,22,24,27,28,29,30,31,57,61]. These have for the most part characterized the bacterial and viral microbiomes separately [14,15,16,24,27,30,31,32,33]. Many have also analyzed pools of ticks. To get a holistic and fine-grained picture of the *I. scapularis* microbiome, we performed unbiased metagenomic sequencing on individual ticks from Wisconsin and quantified the levels of microorganisms using RNA abundance as a proxy for taxon abundance. 

We validated our NGS results by PCR and also found good concordance between our results and those of previous studies of ticks from the same region. The pathogens we detected had all been previously observed in Wisconsin, and our measures of prevalence were in the ranges previously reported [4,62,63,64,65,66,67,68]. For instance, we detected Powassan virus in 1.8% of ticks, while previous studies have detected this virus in 1.3 and 4.6% of ticks [62,63]. We detected *A. phagocytophilum* in 10.7% of ticks, and previous studies have detected this organism in 2.5–14% of ticks [64,66,68]. 

Perhaps the most striking finding of our study was the high prevalence and high relative RNA levels of SoBV and BLTPV (Figure 1). These viruses were both originally identified in *I. scapularis* in New York State, and have been found to be highly prevalent in ticks in several states in the northeastern USA [24,27,30]. Related viruses have also been identified in *I. ricinus* ticks in several European countries [28,29,61]. These studies did not compare levels of these viruses to that of non-viral microbes, and we found that SoBV and BLTPV1 were in fact more abundant and more prevalent than any other members of the microbiota of the ticks we sampled (Figure 2, Figure 3 and Figure 4, Table 1). 

We searched for statistically significant associations in order to identify potential functional interactions between members of the tick microbiota, and also found that SoBV and BLTPV1 were involved in the majority of associations with other organisms. These included a positive correlation between SoBV and *B. burgdorferi* (Figure 9, Supplemental Figure S1). The mechanism by which these viruses might be promoting the replication of other microbes remains unclear. Most studies of the impact of viruses in other arthropods have focused on their ability to interfere with the replication of other viruses [69,70]. Experimental studies will be required to validate these findings and to uncover their mechanistic underpinnings.

Nevertheless, several associations reassured us that our analyses had the potential to detect meaningful relationships. The sole negative interaction was a negative co-occurrence between BLTPV1 and BLTPV2, which were rarely observed in the same tick (Figure 2 and Figure 3, Figure 9). We speculated that this is an instance of superinfection exclusion between relatively closely related viruses (their L segments share ~70% pairwise nucleotide identity). Superinfection exclusion has been documented in other viruses [19,71,72,73,74], including bunyaviruses infecting *Aedes *mosquitoes [75]. We also observed both a positive co-occurrence and a positive correlation between the new filarial worm and *Wolbachia *(Figure 9, Supplemental Figure S1)*. Wolbachia *has been detected previously in *Ixodes* ticks, including a case where the Wolbachia was traced to an infection by an infected endoparasitoid wasp [76]. It is possible that some of the *Wolbachia* sequences we detected were from infections of the tick or some other organisms. However, *Wolbachia *are well-characterized endosymbionts of nematodes [77], and we interpreted the positive co-occurrence and correlation relationships to mean that these were sequences from *Wolbachia *that were infecting worms that were infecting the ticks. As is the case for all hypotheses generated by genomic approaches, these interpretations require experimental validation. Nevertheless, this is an example where shotgun metagenomics provides a richer picture of microbial diversity than would, for example, 16 S sequencing alone [78].

Finally, as has been noted, SoBV, BLTPV, and their relatives have characteristics of mutualistic symbionts [27,79,80]. First, these viruses are highly prevalent in *Ixodes *populations on multiple continents [24,27,28,29,30]. Mutualistic symbionts often manipulate their host’s replication or fitness to favor their own replication, which has the effect of increasing their prevalence in the population [80,81,82]. Second, these viruses have lost their M genome segments, and thus appear to lack a glycoprotein, a typical prerequisite for extracellular enveloped virus infectivity. This has occurred independently in the two lineages, and such genomic contraction has been commonly observed for bacterial endosymbionts during their transition from free-living organisms to obligate heritable symbionts [83]. Lastly, these viruses can be transmitted vertically [27]. Whether these viruses are indeed genuine mutualists remains to be validated experimentally. In fact, apart from their genome sequences, phylogenetic placement, and geographical range, little is known about these viruses. A more in-depth characterization of their biology and biological impact, and that of the tick virome in general, is clearly warranted.

## Figures and Tables

**Table 1:** Taxa prevalence in adult *Ixodes scapularis*.

	Tick Sex		All Ticks		
Taxon	Male	Female	(*n* = 112)	Nucleotide Identity ^1^	Average Mapping Reads Per Million Unique Reads (RPM)
	**(*n* = 51)**	**(*n* = 61)**			
**Viruses**					
Blacklegged tick phlebovirus 1	82.4%	73.8%	77.7%	97.6%	395
South Bay virus	47.1%	55.7%	51.8%	98.0%	2796
Blacklegged tick phlebovirus 2	11.8%	24.6%	18.8%	97.1%	63
Suffolk virus	23.5%	11.5%	17.0%	98.1%	50
Blacklegged tick phlebovirus 3	7.8%	13.1%	10.7%	98.1%	5
Ixodes scapularis associated virus 1	7.8%	1.6%	4.5%	98.3%	3
Powassan virus	0%	3.3%	1.8%	100.0%	55
Ixodes scapularis associated virus 2	2.0%	0%	0.9%	96.4%	2
**Bacteria**					
*Rickettsia* spp.	5.9%	80.3%	46.4%	98.8%	21
*Borreliella burgdorferi*	33.3%	47.5%	41.1%	99.7%	154
*Anaplasma phagocytophilum*	11.8%	9.8%	10.7%	98.9%	28
*Wolbachia* spp.	5.9%	9.8%	8.0%	97.5%	10
*Ehrlichia* spp.	5.9%	1.6%	3.6%	96.6%	45
*Borrelia miyamotoi*	0%	3.3%	1.8%	100.0%	64
*Borrelia mayonii*	0%	1.6%	0.9%	99.4%	1
**Eukaryotes**					
Novel filarial worm	21.6%	14.8%	17.9%	88.7%	113
*Babesia odocoilei*	5.9%	13.1%	9.8%	100.0%	22
*Babesia microti*	3.9%	13.1%	8.9%	99.7%	52

^1^ Mean identity of BLASTn alignments of contigs to closest related database sequences.

**Table 2:** New virus-like sequences identified.

	Closest Related Sequence	Contig Length (nt)	Accession	BLAST Percent Identity ^1^
Norway mononegavirus 1-like sequence	Norway mononegavirus 1 (MF141072.1)	5020	MH560586	71%
Blacklegged tick phlebovirus-like sequence	Blacklegged tick phlebovirus 1 (KX184201.1)	874	MH560584	77%
Norway phlebovirus 1-like sequence	Norway phlebovirus 1 (MF141061.1)	791	MH560585	78%

^1^ Pairwise identity of BLASTn alignment to highest scoring database sequence.

## Supplementary Materials

The following are available online at http://www.mdpi.com/1999-4915/10/7/388/s1.
