# Supplementary material for: Co-Infection Patterns in Individual Ixodes scapularis Ticks Reveal Associations between Viral, Eukaryotic and Bacterial Microorganisms
Source: Viruses. 2018 Jul 22;10(7):388. doi: 10.3390/v10070388 (PMC6071216; doi:10.3390/v10070388)
Supplement: Supplementary file 1 [file viruses-10-00388-s001.zip › suppl_fig_1_abundance_scatterplot.pdf]

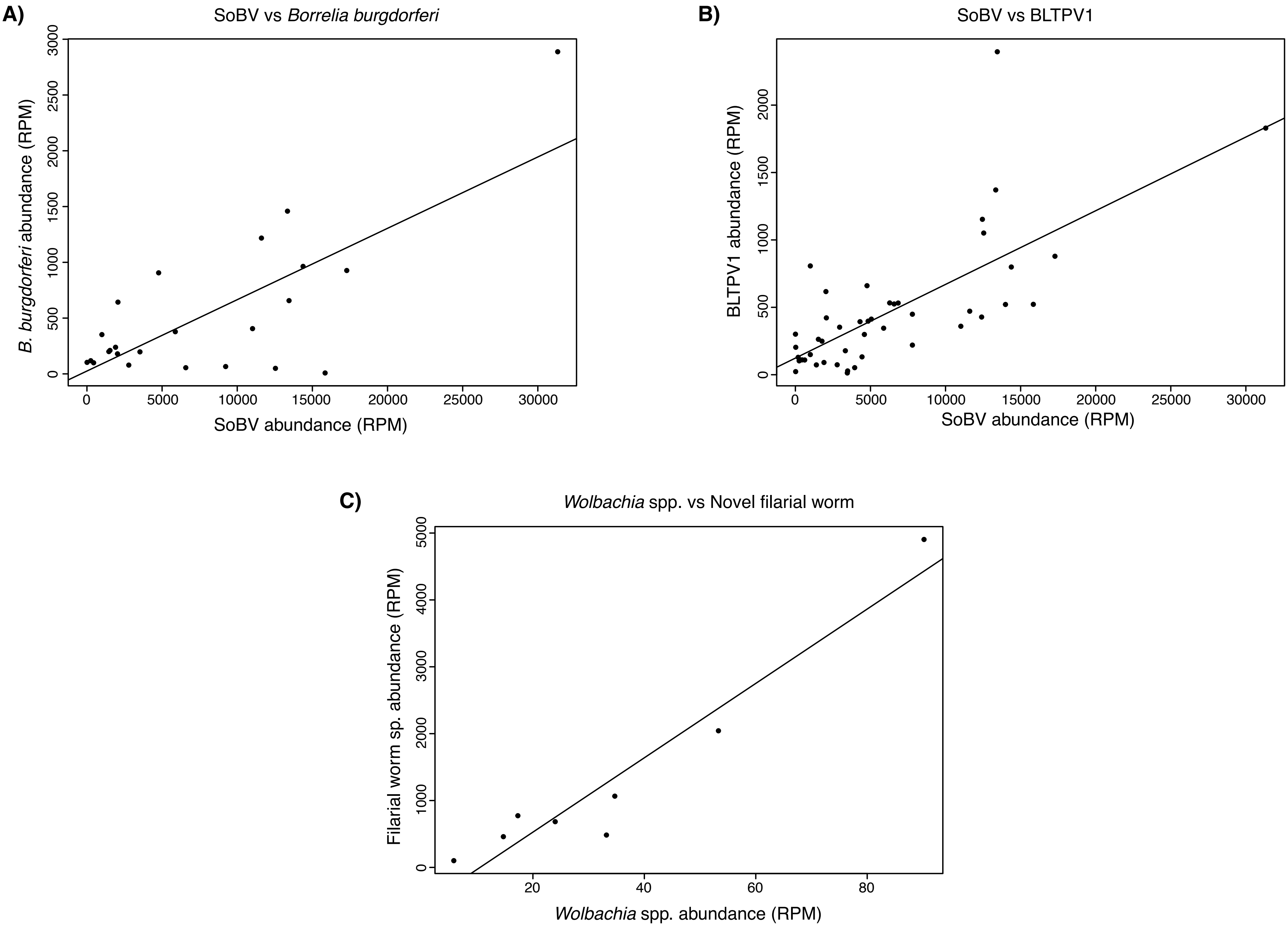

Supplemental Figure 1. Positive correlations are seen within multiple microbial pairs by abundance. Scatterplot of RNA levels (mapping reads per million unique reads; RPM) for (a) South Bay virus (SoBV) and *Borrelia burgdorferi*, (b) SoBV and blacklegged tick phlebovirus 1 (BLTPV1), and (c) *Wolbachia* spp. and the novel filarial worm specie. Linear regression lines are also plotted.
